# Supplementary material for: Behavioral representational similarity analysis reveals how episodic learning is influenced by and reshapes semantic memory
Source: Nat Commun. 2023 Nov 20;14:7548. doi: 10.1038/s41467-023-42770-w (PMC10662157; doi:10.1038/s41467-023-42770-w)
Supplement: Supplementary file 1 — Supplementary Information [file 41467_2023_42770_MOESM1_ESM.pdf]

Supplementary Methods

Determining our sample size

Meta-analysis of testing effect studies reveals that the advantage of tested words pairs over restudied word pairs on final memory performance is typically a medium size effect, with a mean effect size of g = 0.50 across 159 effect sizes from 61 studies[1]. When restricting the meta-analysis to studies that only included semantically unrelated pairs (g = 0.67) or studies that only included semantically related pairs (g = 0.66), it appears that the size of the testing effect is not heavily impacted by semantic relatedness. However, there is limited literature on the topic, so individual empirical studies might be more informative than a larger meta-analysis[1]. One particularly relevant study[2] directly compared semantically related triads to semantically unrelated triads in a repeated measures testing effect design with multiple cycles of a study/test manipulation. They found that relatedness had an effect size of d = 2.94 after one test and an effect size of d = 2.58 after two tests, both of which constitute large effect sizes. They also reported a significant learning condition (test vs restudy) x relatedness interaction effect size of d = 0.55, suggesting a medium effect size.

Although the literature suggests medium to large effect sizes for the impact of our learning condition and semantic relatedness manipulations on final test performance, we anticipated that the effect of these manipulations on our representational similarity measures would be considerably smaller (i.e. learning-induced perturbations of semantic space should be subtle, given the overall stability of participants' pre-experimental semantic knowledge). Since there was nothing in the literature to anchor our effect size calculations for our novel representational similarity approach, we assumed a Cohen's d of 0.2 (a small effect size) when calculating our target sample size. Using this effect size, we reasoned that we would need a sample size of at least 73 useable participants to reach a power of 0.80 for the learning condition x relatedness interaction (calculations performed using PANGEA (v0.2); https://jakewestfall.shinyapps.io/pangea/). As such, we aimed to collect useable data from 80 participants.

Inclusion/Exclusion criteria:

Recruitment was restricted to adults (age 18-40) from the United States, Canada, and Mexico, whose first language is English, who have no current or ongoing mental health or neurological condition and who had successfully completed a minimum of 10 prior studies on Prolific.

Additionally, participants who failed to meet the following pre-registered criteria were excluded:

- Fail to complete Day 2 within 28 hours of completing Day 1
- Fail to respond correctly to attention checks
- Have a median RT of less than 500 ms in the first learning session while making relatedness judgements
- Fail to show a difference between relatedness judgements on related and unrelated words
- Report that they believe their data should be excluded or report some sort of technical issue
- Report distraction of greater than 5 out of 7 on our distraction scale for either of the days, or report three or more distractions occurring on either day
- Do not perform a meaningful arrangement (see below for details) on one or more of the trials of the word arrangement task
- Do not have a sufficient performance on the memory tasks. For example, the participants will be excluded if any of the following are true:
    - Those who fail to correctly retype three or more of the restudy trials on the initial learning day

  - Those who fail to recall more than 25% of the tested associates during the initial learning session, or more than 25% across all pairs during the second session
- Explicitly report writing pairs down between sessions

Additionally, initial and final arrangements following imputation were visually inspected to determine compliance with the task. Participants who judged similarity based on lexical characteristics (i.e. by the first letter of each word) or who did not use any meaningful arrangement and randomly placed words on the page, were excluded. Meaningfulness was judged by three independent raters, and participants were excluded if at least two of the three raters agreed that either arrangement was considered not meaningful.

R Session Information

R version 4.2.2 (2022-10-31)
Platform: aarch65-apple-darwin20 (64-bit)
Running under: macOS Monterey 12.4

Other attached packages and versions:

- effectsize (0.8.3)
- emmeans (1.8.3)
- varTestnlme (1.3.1)
- r2mlm (0.3.2)
- nlme (3.1-160)
- forcats (0.5.2)
- stringr (1.5.0)
- dplyr (1.1.0)
- purrr (1.0.1)
- readr (2.1.4)
- tidyr (1.3.0)
- tibble (3.2.1)
- ggplot2 (3.4.2)
- tidyverse (1.3.2)
- rstatix (0.7.1)
- reshape2 (1.4.4)
- lmerTest (3.1-3)
- lme4 (1.1-31)
- Matrix (1.5-0)
- corrplot (0.92)
- ggpattern (1.0.1)
- easystats (0.6.0)

Supplementary Note 1

Behavioral Judgements of Relatedness

On average, participants rated semantically related pairs (M = 3.737, SD = 0.578) as more similar to each other than semantically unrelated pairs (M = 1.559, SD = 0.780) ($F_{(1,79)} = 3328.743$, $p < 0.001$, $\eta_G^2 = 0.941$). Similarity, participants rated semantically related pairs (M = 3.711, SD = 0.609) more likely to be found on the same page of a book or magazine than semantically unrelated pairs (M = 1.676, SD = 0.864) ($F_{(1,77)} = 1704.76$, $p < 0.001$, $\eta_G^2 = 0.896$). Judgements across questions were significantly correlated with each other ($r_{(4394)} = 0.854$, $p < 0.001$, 95% CI=[0.846 0.862]), as well as with word2vec similarity (relatedness judgement: $r_{(4550)} = 0.710$, $p < 0.001$, 95% CI=[0.696 0.724]; page judgement: $r_{(4533)} = 0.675$; $p < 0.001$, 95% CI=[0.658 0.690]) and LSA (relatedness judgement: $r_{(4550)} = 0.718$; $p < 0.001$, 95% CI=[0.703 0.732]; page judgement: $r_{(4533)} = 0.675$; $p < 0.001$, 95% CI=[0.659 0.691]). Results are visualized in Supplementary Figure 1.

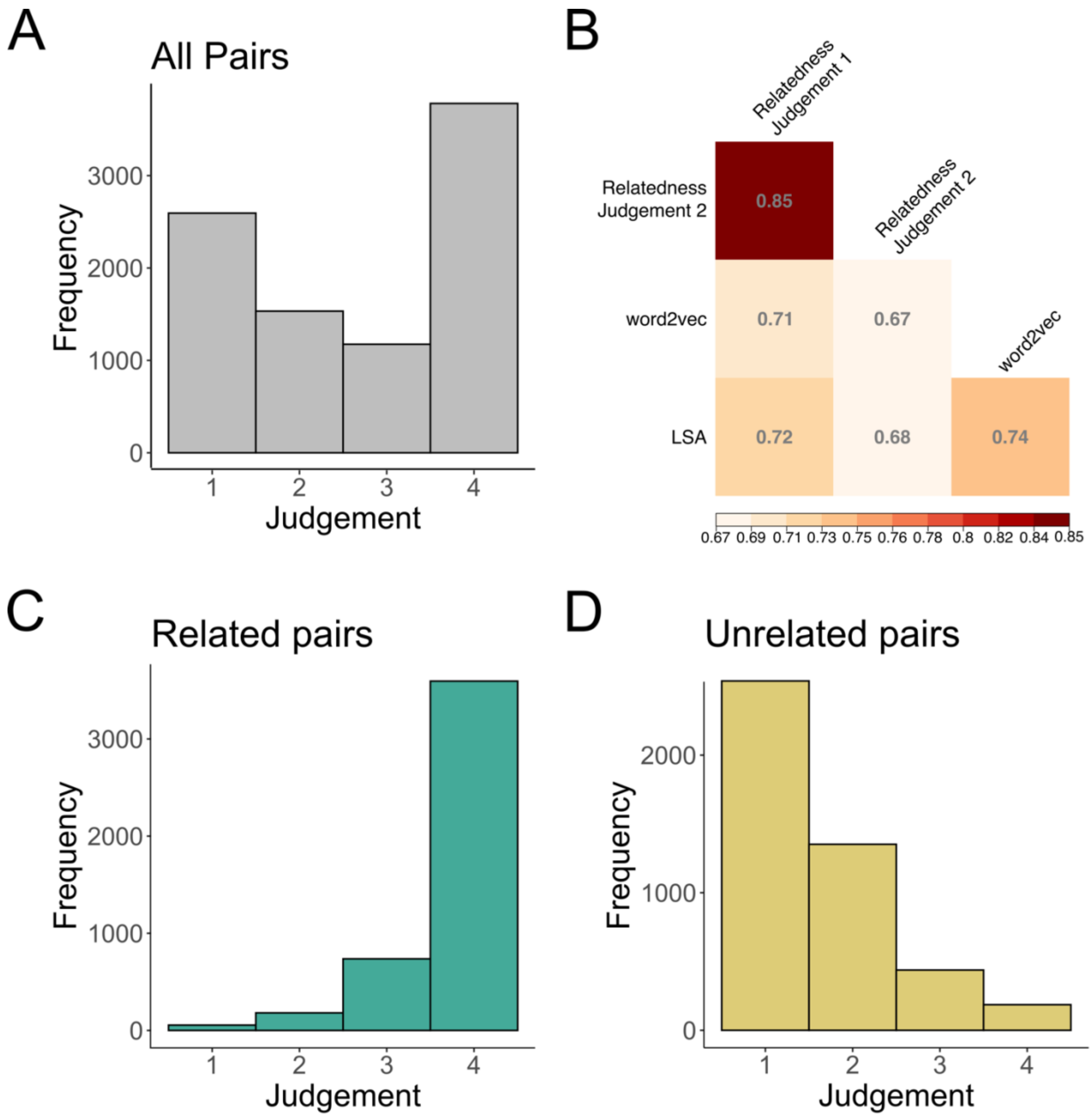

Supplementary Figure 1: Behavioral Judgements of Relatedness During Learning. A. Histogram of all relatedness judgements during learning. B. Correlations of participants' relatedness judgements to normative measures of semantic relatedness. C/D. Histogram of relatedness judgements for semantically related and semantically unrelated pairs. Data come from 9,093 judgments across N = 80 participants

Memorability of Word Pairs

The memorability of each word pair was operationalized by measuring the average accuracy for each word pair across subjects. There was a wide range of memorability across word pairs (Supplementary Figure 2; M = 0.455, SD = 0.233, range 0.05 – 0.913). When average accuracy was entered into a between subjects ANOVA, there was no statistically significant main effect of word set ($F_{(1,116)} = 0.623$, $p = 0.432$, $\eta_G^2 = 0.005$) and no statistically significant interaction of word set and learning condition ($F_{(1,116)} = 0.226$, $p = 0.635$, $\eta_G^2 = 0.002$).

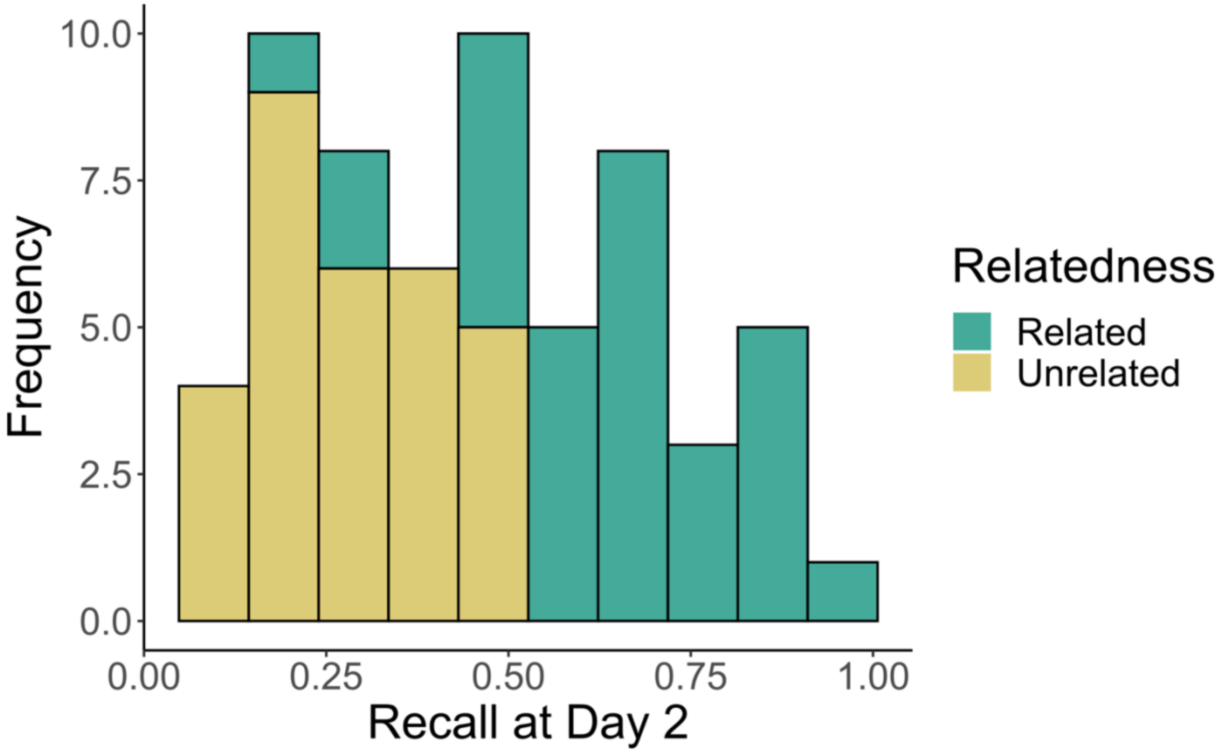

Supplementary Figure 2: Distribution of memorability across word pairs. Memorability was operationalized by computing the average recall accuracy across participants and learning condition. Memorability ranged from 0.05 - 0.913. Despite the wide range of memorability, there was no statistically significant difference in memorability across word pair sets used to randomize learning condition ($F_{(1,116)} = 0.623$, $p = 0.432$, $\eta_G^2 = 0.005$) or interaction with learning condition ($F_{(1,116)} = 0.226$, $p = 0.635$, $\eta_G^2 = 0.002$). N = 60 pairs of words across N=80 individual participants. Green reflects semantically related word pairs, yellow reflects semantically unrelated word pairs.

Investigating Impact of Task Switching

One potential confound to our behavioral results could stem from task-switching during the third learning opportunity (where our learning condition manipulation was introduced). Our test condition (where participants had to actively retrieve and type a cued target word from memory) could be considered quite different from our restudy condition (where participants were asked to restudy the pair by re-typing the presented target word). Because test and restudy trials were intermixed, there was necessarily some degree of task switching each time participants encountered a trial of a different condition than the prior trial. Given the added cognitive load associated with a task switch, it is possible that switch trials would have different learning outcomes than non-switch trials. To test for the potential impact of task switching during learning, we characterized each trial as either a switch trial (where the learning condition was different from the previous trial; i.e. $trial_{t-1}$ was a test trial and $trial_t$ was a restudy trial) or a non-switch

trial (where the learning condition was consistent with the prior trial; i.e. both trial$_{t-1}$ and trial$_t$ were restudy trials) and included this trial type as a factor with learning condition and semantic relatedness in a RM-ANOVA on the behavioral accuracy at Day 2. Regardless of whether or not we bifurcated the test trials into those correctly and incorrectly recalled at Day 1, there was no statistically significant main effect of trial type (switch trial vs non-switch trial; non-bifurcated: $F_{(1, 79)} = 0.061$, $p = 0.806$, $\eta_G^2 = 3.94 \times 10^{-5}$; bifurcated: $F_{(1,44)} = 0.828$, $p = 0.368$, $\eta_G^2 = 0.001$), and trial type did not significantly interact with either of our other factors (all p-values > 0.05; Supplementary Table 1).

**Effect**	**DFn**	**DFd**	**F**	**p**	η_G^2
Learning Condition	2	88	238.398	< 0.001	0.505
Relatedness	1	44	166.275	< 0.001	0.236
Trial Type	1	44	0.828	0.368	0.001
Learning Condition x Relatedness	2	88	9.581	< 0.001	0.028
Learning Condition x Trial Type	2	88	2.463	0.091	0.006
Relatedness x Trial Type	1	44	2.798	0.101	0.005
Learning Condition x Relatedness x Trial Type	1.59	69.9	0.268	0.714	0.000748

Supplementary Table 1: RM-ANOVA results testing the impact of trial type (switch trial vs non-switch trial) when tested trials are bifurcated into those are recalled correctly at Day 1 and those that are incorrectly recalled at Day 1. Data are from N=80 participants and all statistical tests are two-tailed. No corrections for multiple comparisons were performed.

Supplementary Note 2

Validation of Imputation

Given that one of our main dependent variables in our analyses is the change in dissimilarity/similarity before and after learning, it is imperative to validate that our imputed values are a meaningful approximation of what they would be if they were experimentally measured. First, we sought to determine the appropriate number of nearest neighbors in our imputation algorithm. To do so, we artificially 'lesioned' a random sample of observed pairwise dissimilarity values obtained from an independent sample of pilot participants, and we varied the number of neighbors used from 1 to 50 to impute the missing ('lesioned') values. For each number of neighbors, the Pearson correlation of the imputed dissimilarity value and the true measured dissimilarity value was calculated, as well as the mean error between the imputed and measured dissimilarity values. A final value of 40 neighbors was chosen, as it maximized the correlation between true and imputed value and minimized error.

Once an optimal number of neighbors was identified from our pilot sample, we turned to our measured data and lesioned a new set of 60 dissimilarity measures from our raw, unimputed data. Although we are theoretically only interested in imputing 60 pre-defined pairs of interest, our actual imputation task requires the imputation of large "blocks" of missing data in the similarity matrix. Lesioning additional values from our unimputed behavioral dataset (rather than 60 uncorrelated values from a fully measured matrix) ensured that the validation imputation process would have access to the approximately the same amount of data as in the final imputation process and equated the total amount of missing data across the validation and true procedure. The lesioned data were imputed, and the new imputed values were correlated with the true values. We also obtained the normative similarity of each to-be-learned pair from word2vec, and then correlated these values with the imputed dissimilarity scores. There were significant correlations between the lesioned and measured values before learning ($r_{(58)} = 0.92$, $p < 0.001$, 95% CI =

[0.85 0.95]) and after learning ($r_{(58)} = 0.92$, $p < 0.001$, 95% CI = [0.87 0.95])) (Supplementary Figure 3A-B).

We additionally repeated this procedure on semantic space as measured by word2vec. Because the semantic space from word2vec did not include the blocks of missing data that our measured similarity did, we first removed the similarity values that would be missing from the measured data to equate amount of missing data in the imputation. We then artificially lesioned an additional 60 pairs (as was done in the previous validation analyses), imputed the lesioned word2vec values and compared the imputed values to the true values. Just as in the measured behavioral data, there was a significant correlation between the imputed and true values ($r_{(58)} = 0.65$, $p<0.001$, 95% CI = [0.47 0.77]) (Supplementary Figure 3C). Additionally, we compared imputed similarity values to similarity values derived from word2vec corpus. This showed a significant correlation ($r_{(58)} = 0.61$, $p < 0.001$, 95% CI=[0.428 0.751]) (Supplementary Figure 3D).

An alternative way to approach measuring similarity for a pair of words A and B would be to identify the set of 90 words that both word A and word B were measured against. It would then be possible to operationalize word A and word B by a 90-dimension vector containing those shared values. Concretely, even if the pair GENDER – FEMALE was never directly measured, we could operationalize both GENDER and FEMALE by their distance from the words PARENT, MOTHER, and TEACHER (among the other words that GENDER and FEMALE were both compared against). We could then measure similarity by computing the correlation between the two 90-dimension vectors. There was a significant correlation between the Fisher z-transformed correlation values from the shared vector approach similarity and the imputed similarity of our to-be-learned pairs for both the initial ($r_{(4558)} = 0.63$, $p < 0.001$, 95% CI=[0.616 0.650]) (Supplementary Figure 3E) and final arrangements initial ($r_{(4558)} = 0.71$, $p < 0.001$, 95% CI = [0.690 0.720]) (Supplementary Figure 3F). This shared vector approach has the benefit of avoiding imputation entirely; future work may explore whether there are optimal conditions for choosing one approach over the other, or whether the approaches perform similarly.

Finally, a linear mixed model was used to compare imputed dissimilarity across participants' explicit behavioral judgements of similarity from the learning phase. Linear mixed modeling showed that imputed dissimilarity was significantly different across relatedness judgements for both initial arrangement ($F_{(3,4538)} = 42.43$, $p < 0.001$, $\eta_p^2 = 0.03$) and final arrangement ($F_{(3,4539)} = 68.87$, $p < 0.001$, $\eta_p^2 = 0.04$) (Supplementary Figure 4).

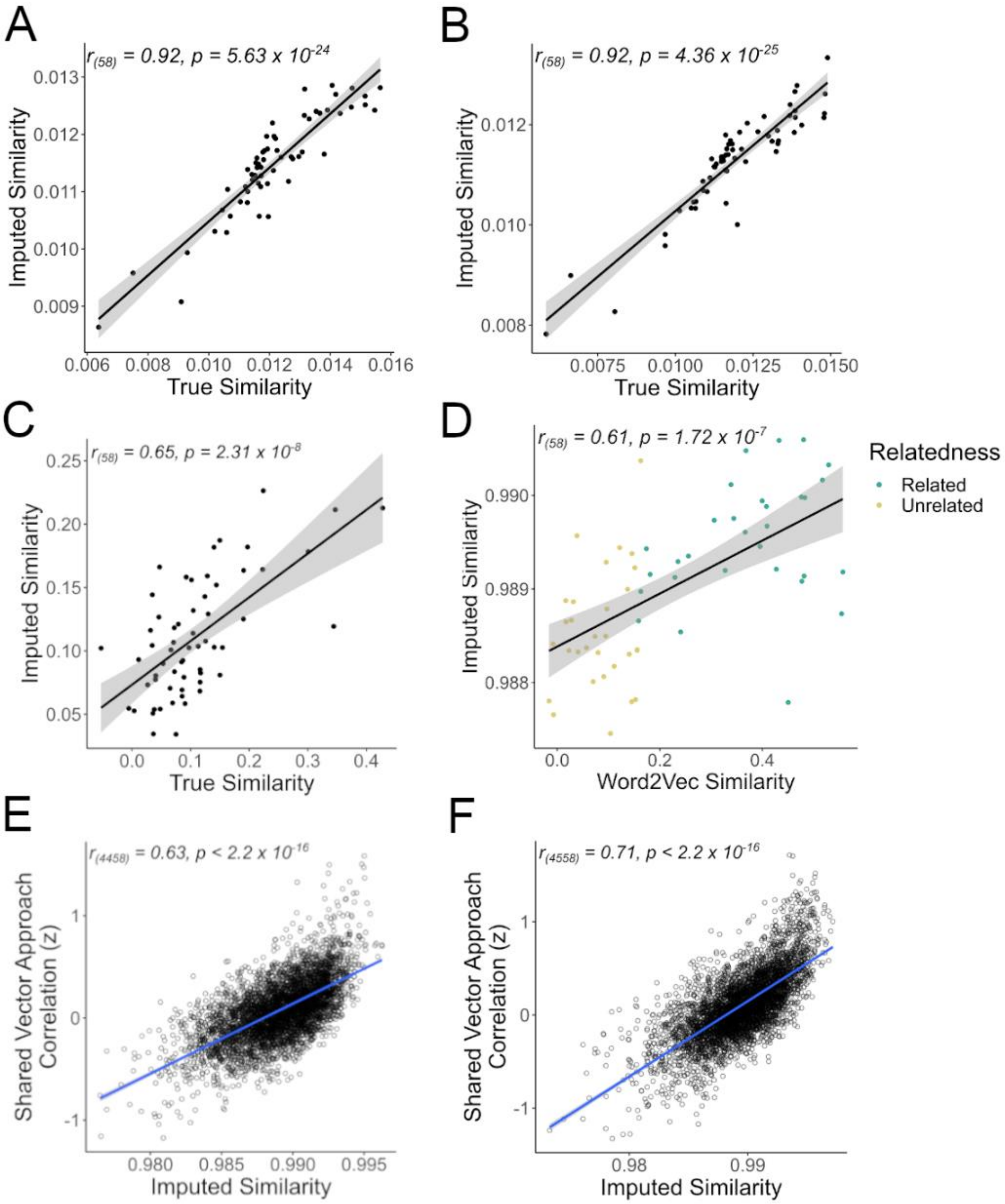

Supplementary Figure 3: Comparing imputed similarity to measured values. A-C Imputed and measured similarity from a “lesioned” semantic space showed significant correlations before learning (A: $r_{(58)} = 0.92$, $p < 0.001$, CI = [0.85 0.95]), after learning (B: $r_{(58)} = 0.92$, $p < 0.001$, CI = [0.87 0.95])), and when the procedure was performed on normative semantic space as measured by word2vec (C: $r_{(58)} = 0.65$, $p < 0.001$, CI = [0.47 0.77])). D. Imputed similarity was significantly correlated ($r_{(58)} = 0.61$, $p < 0.001$, CI = [0.428 0.751])) with similarity ratings derived from word2vec. Each dot reflects a single similarity score for a pair of words. E-F. Imputed similarity showed significant correlations to a shared vector similarity approach both before learning (E: $r_{(4458)} = 0.63$, $p < 0.001$, CI = [0.616 0.650])) and after learning (F: $r_{(4458)} = 0.71$, $p < 0.001$, CI = [0.690 0.720]). For A-D, N = 60 word pairs. For E and F, N = 4,560 word pairs across 80 participants. Green represents semantically related pairs, yellow semantically unrelated pairs. Shaded area reflects 95% CI.

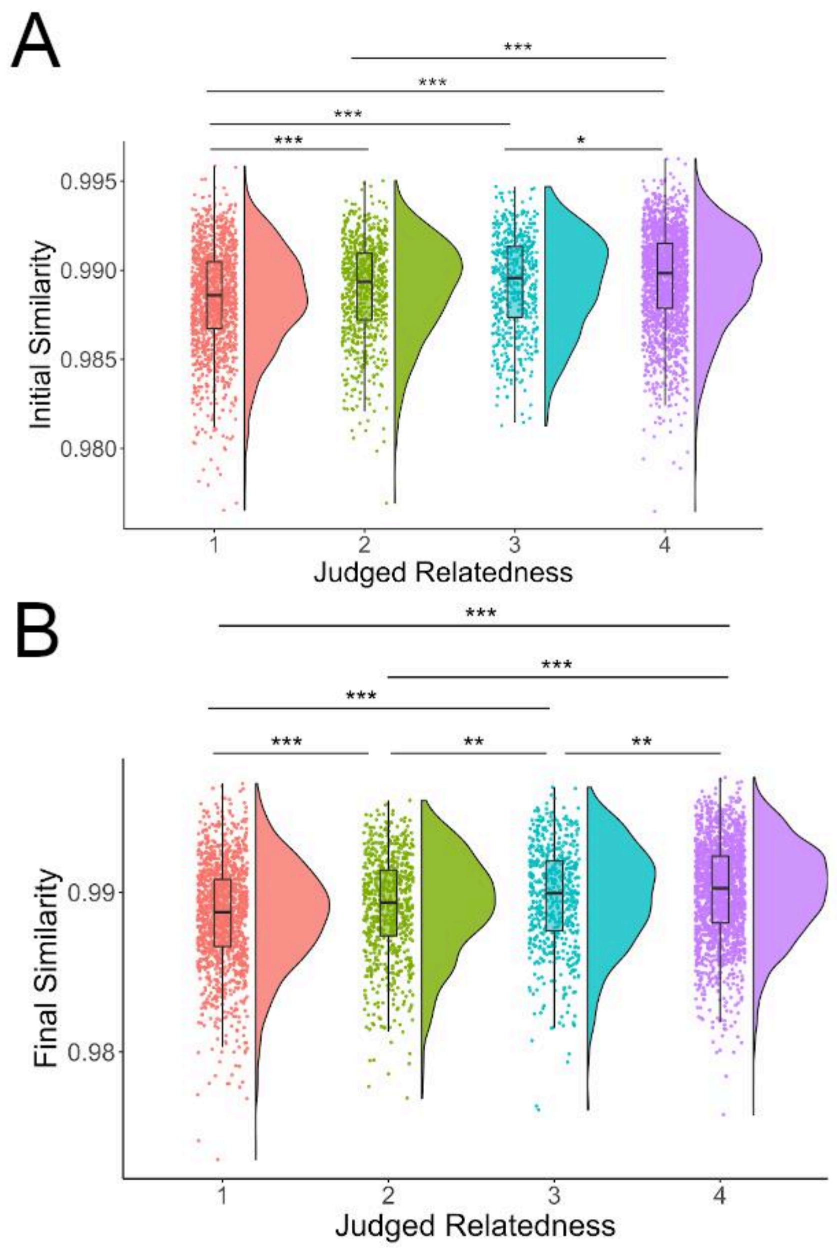

Supplementary Figure 4: Comparing imputed similarity to relatedness judgements. Linear mixed modeling showed that imputed similarity was significantly different across relatedness judgements for both the initial arrangement (A: $F_{(3,4538)} = 42.43$, $p < 0.001$, $\eta_p^2 = 0.03$) and final arrangement (B; $F_{(3,4539)} = 68.87$, $p < 0.001$, $\eta_p^2 = 0.04$). N = 4,550 imputed similarity scores from 80 independent participants. Dots reflect an individual imputed similarity score. Boxplots reflect median, 25th and 75th percentiles; whiskers show 95% CI. Symbols reflect statistically significant differences across conditions using Holm-Bonferroni corrections for multiple comparisons (* p < 0.05, ** p < 0.01, *** p < 0.001).

Supplementary Note 3

Change in Similarity – To-Be-Learned Pairs

In our main analyses, we showed that the pairwise change in similarity in our conditions of interest were significantly different from zero and report a linear mixed-effects modeling (LMM) approach to show that there were no differences in the amount of pairwise change in similarity across relatedness and learning condition. However, we pre-registered that we would test hypotheses surrounding the differences in pairwise change of similarity using an RM-ANOVA approach, so results of those analyses are included here for completeness. Using an RM-ANOVA on the change in similarity across learning showed no statistically significant main effect for relatedness ($F_{(1,49)} = 1.453$, $p = 0.234$, $\eta_G^2 = 0.004$), no statistically significant main effect for learning condition ($F_{(1,49)} = 0.415$, $p = 0.523$, $\eta_G^2 = 0.001$), and no statistically significant relatedness by learning condition interaction ($F_{(1,49)} = 0.394$, $p = 0.533$, $\eta_G^2 = 0.001$). These results are consistent with those from an LMM framework reported in the main text.

Relatedness	Learning Condition	Correct at Final?	M	95% CI	t	df	p	Cohen's d
Related	Restudy	No	0.00050	[0.00005 0.00095]	2.226	77	0.145	0.252
Related	Restudy	Yes	0.00071	[0.00038 0.00104]	4.258	79	< 0.001	0.476
Related	Test	No	0.00019	[-0.00047 0.00086]	0.584	64	1.000	0.072
Related	Test	Yes	0.00064	[0.0003 0.00098]	3.788	79	0.002	0.423
Unrelated	Restudy	No	0.00013	[-0.00015 0.00042]	0.933	79	1.000	0.104
Unrelated	Restudy	Yes	0.00019	[-0.00043 0.00081]	0.616	73	1.000	0.072
Unrelated	Test	No	0.00012	[-0.00052 0.00075]	0.365	72	1.000	0.043
Unrelated	Test	Yes	0.00077	[0.00027 0.00127]	3.085	74	0.017	0.356

Supplementary Table 2: Two-tailed one sample t-tests testing whether the change in similarity of word pairs is different from zero. Data come from N=80 participants and the p-values are corrected for multiple comparisons using the Holm-Bonferroni method.

Change in Similarity – Lures

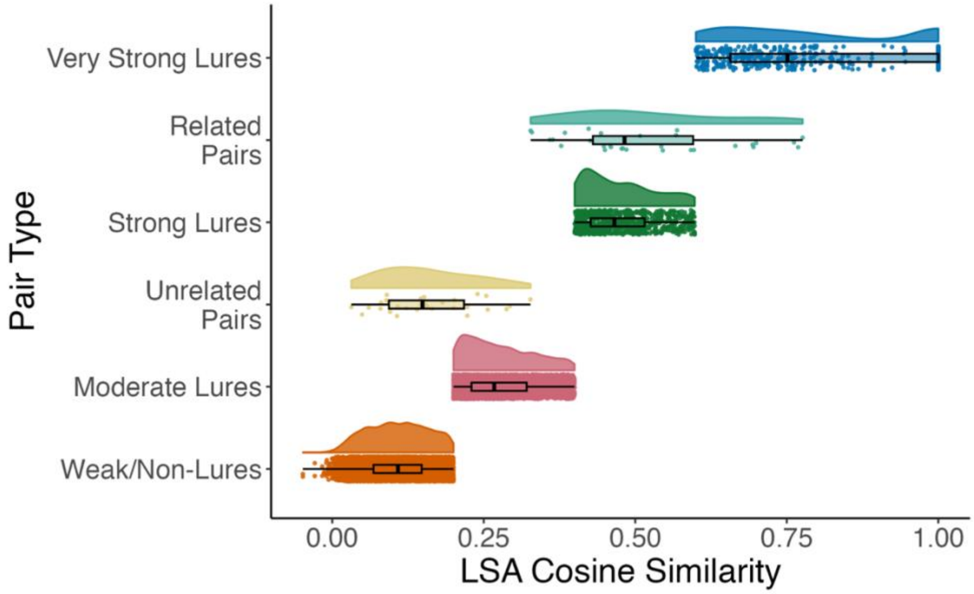

Supplementary Figure 5: Distribution of LSA cosine similarity of to-be-learned pairs and potential lure pairs. LSA cosine similarity was computed for all related (N = 30 pairs; M = 0.52, SD = 0.13; light green) and unrelated (N = 30 pairs; M = 0.15, SD = 0.8; yellow) to be learned pairs of words. Lure pairs (i.e. all 118 potential word pair combinations for the 60 to-be-learned cues) were each defined as a non-lure/weak lure (LSA < 0.2; n = 9,056 pairs, M = 0.11, SD = 0.05; orange), moderate lure (LSA between 0.2 and 0.4; n = 4011 pairs, M = 0.28, SD = 0.06; pink), strong lure (LSA between 0.4 and 0.6; n = 844 pairs, M = 0.48, SD = 0.06; dark green) and very strong lure (LSA above 0.6; n = 429, M = 0.79, SD = 0.06; blue). Dots reflect the LSA cosine similarity value for a given pair of words in our corpus. Boxplots reflect median, 25th and 75th percentiles; whiskers show 95% CI.

We first tested whether the lures across strength bins and learning conditions showed change that was significantly different from zero. To that end, we computed two-tailed one-sample t-test (with Holm-Bonferroni corrections for multiple comparisons) to determine whether there was change in semantic similarity across learning. For lures associated with restudied pairs, weak/non-lures ($t_{(73369)}$=4.815, p<0.001, Cohen's d=0.018, 95% CI=[7.565x10^{-5} 1.795x10^{-4}]), moderate lures ($t_{(33934)}$=3.786, p<0.001, Cohen's d=0.02, 95% CI=[7.299x10^{-5} 2.297x10^{-4}]), strong lures ($t_{(7861)}$=3.929, p< 0.001, Cohen's d=0.04, 95% CI=[1.642x10^{-4} 4.913x10^{-4}]), and very strong lures ($t_{(2242)}$=8.234, p<0.001, Cohen's d=0.17, 95% CI=[9.105x10^{-4} 1.479x10^{-3}]) were drawn closer together after learning.

For lures associated with tested pairs, weak/non-lures ($t_{(799776)}$=5.726, p<0.001, Cohen's d=0.02, 95% CI=[9.515x10^{-5} 1.942x10^{-4}]), strong lures ($t_{(9791)}$=4.904, p<0.001, Cohen's d=0.05, 95% CI = [2.203x10-4 5.136x10-4]), and very strong lures ($t_{(2849)}$=7.918, p<0.001, Cohen's d=0.15, 95% CI=[7.407x10^{-4} 1.228x10^{-3}]) were significantly drawn together after learning; the change of moderate lures associated with tested pairs was not significantly different from zero ($t_{(38679)}$=-0.438, p=0.661, Cohen's d=0.002, 95% CI=[2.203x10^{-4} 5.136x10^{-4}]).

Next, we tested the pairwise comparisons of all lure strength bins across learning condition (i.e. the lure strength by learning condition interaction). To do so, we used a series of two-tailed paired t-tests with Holm-Bonferroni corrections for multiple comparisons. In addition to the results reported in the main text, we showed that for restudied pairs, the representations of very strong lures are drawn towards cue words more so than strong lures (z=5.068, p< 0.001, Cohen's d=0.13, 95% CI=[-1.38x10^{-3} -4.50x10^{-4}]) and moderate lures (z=6.624, p<0.001, Cohen's d=0.15, 95% CI=[-1.5x10^{-3} -6.61x10^{-4}]) are drawn towards cue words. For tested pairs, very strong lures show a similar pattern where they are drawn towards cue words more so than strong lures (z=4.326, p<0.001, Cohen's d=0.10, 95% CI=[-1.10x10^{-3} -2.80x10^{-4}]) and moderate lures (z=7.260, p<0.001, Cohen's d=0.15, 95% CI=[-1.42x10^{-3} -6.76x10^{-4}]) are drawn towards cue words; additionally, tested strong lures are also drawn towards cue words more so than tested moderate lures (z=4.090, p< 0.001, Cohen's d=0.05, 95% CI=[-5.81x10^{-4} -1.33x10^{-4}]).

In addition to the analyses of the pairwise differences in change in similarity across lure bins, we also tested the difference across learning condition in very strong, strong, and moderate lures relative to a weak/non-lure baseline. In the main text, we report that moderate lures associated with tested pairs are drawn together less than those associated with restudied pairs (z=2.840, p=0.014, d=0.03, 95% CI=[5.740x10^{-5} 3.129x10^{-4}]). Neither strong lures (z = 0.049, p=0.961, d=0.0008, 95% CI = [-0.0002 0.0002]) nor very strong lures (z = -1.052, p = 0.585, d=-0.030, 95% CI = [-0.0006 0.0002]) showed statistically significant differences across learning condition.

Supplementary Note 4

Representational change relative to normative space

Another way to test for differential representational change of cue and target words is to compare each word's representation following learning to a normative semantic template. The observed changes can provide insight into how the representational changes relate to a more general, normative structure. That

is, we can test whether the changes result from sculpting existing semantic information or by creating idiosyncratic elaborative connections that would not be captured by a normative representation. To do so, each similarity vector (defined by the 20 closest nearest neighbors, as was reported in analyses in the main text) was correlated with its respective similarity vector derived from word2vec. This value was Fisher z-transformed and the difference in correlation across learning was entered into an LMM. As with the previous analyses in the main text, the semantic relatedness of the pair, learning condition, recall success at final test and word's position in the to-be-learned pair were included as a fixed effect predictors and tested as potential random slopes. Ultimately, no random slopes were included and only subject identity was included as a random intercept.

This model showed a significant main effect of learning condition ($t_{(1137)} = 2.211$, p = 0.027, Cohen's d = 0.07, 95% CI=[0.002 0.039]), where tested pairs showed more change than restudied pairs; Supplementary Figure 6. No other predictors were statistically significant (word position: $t_{(1126)} = 0.100$, p = 0.920, Cohen's d = 2.98×10^{-3}, 95% CI = [-0.017 0.019]; relatedness: $t_{(1137)} = -0.627$, p = 0.531, Cohen's d = 0.02, 95% CI = [-0.024 0.012]; success at final recall: $t_{(1139)} = -0.198$, p = 0.844, Cohen's d = 5.87×10^{-3}, 95% CI = [-0.020 0.016]). Taken together with our results from the main text—where we show that strongly related lures are pulled closer to the to-be-learned cue and that testing pushes moderately related lures further away relative to the baseline general changes to semantic space—these results suggest that testing may shape existing features of the paired concepts, rather than adding new features to the representation, and that this process occurs for both cue and target words.

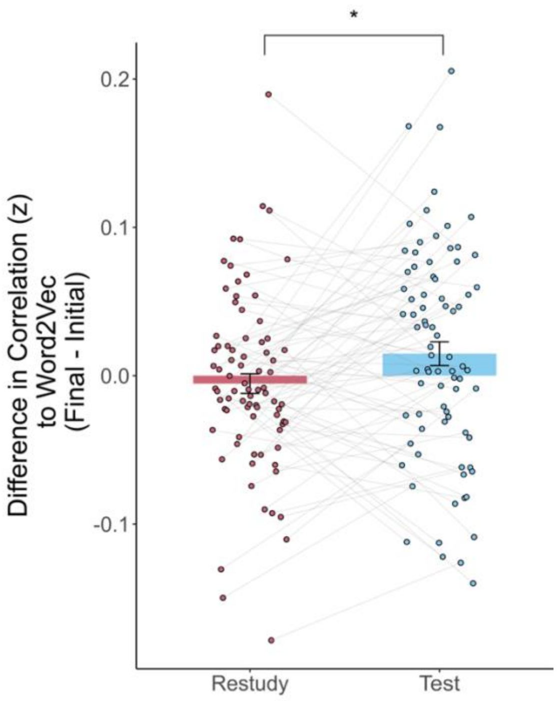

Supplementary Figure 6: Relating idiosyncratic semantic structure to normative semantic space. Words in tested pairs, regardless of word position in pair or semantic relatedness of pair, showed more change relative to analogous normative semantic representations derived from word2vec ($t_{(1137)} = 2.211$, $p = 0.027$, Cohen's d = 0.07, 95% CI=[0.002 0.039]). All displayed correlation values are Fisher *r*-to-*z* transformed. Maroon bar reflects restudied pairs, blue bar reflects tested pairs. Open circles reflect means of N=80 individual participants, with connecting lines showing within-participant differences across conditions. Error bars reflect standard error of the mean. Symbols reflect statistically significant differences across conditions using Holm-Bonferroni corrections for multiple comparisons (* $p < 0.05$).

Supplementary References

1. Rowland, C. A. The Effect of Testing Versus Restudy on Retention : A Meta-Analytic Review of the Testing Effect. *Psychol. Bull.* (2014) doi:10.1037/a0037559.

2. Bulevich, J. B., Thomas, A. K. & Parsow, C. Filling in the gaps: using testing and restudy to promote associative learning. *Memory* **24**, 1267–1277 (2016).

3. Storm, B. C., Friedman, M. C., Murayama, K. & Bjork, R. A. On the transfer of prior tests or study events to subsequent study. *J. Exp. Psychol. Learn. Mem. Cogn.* **40**, 115–124 (2014).

Pairwise Change in Similarity of To-Be-Learned Pairs

Fixed Effects

	Coefficient	*t-value*	*S.E.*	*p-value*
Memory Success at Day 2	0.0003*	1.965	0.0002	0.0499
Learning Condition	0.00006	0.3933	0.0002	0.6942
Relatedness	-0.0002	-1.3161	0.0002	0.1887
Lure Strength - Weak Lures				
Lure Strength - Moderate Lures				
Lure Strength - Strong Lures				
Relatedness x Lure Strength - Weak Lures				
Relatedness x Lure Strength - Moderate Lures				
Relatedness x Lure Strength - Strong Lures				
Learning Condition x Lure Strength - Weak Lures				
Learning Condition x Lure Strength - Moderate Lures				
Learning Condition x Lure Strength - Strong Lures				
Learning Condition x Relatedness				
Num.Obs.	605			
AIC	-5685.6			
BIC	-5659.2			
ICC	0.1			
RMSE	0.002			

Random Effects

	Variable	Variance	Std. Dev
PTID	(Intercept)	4.89E-07	0.00069945
Residual		3.96E-06	0.00198879

Pairwise Change in Similarity of Semantic Lures

Fixed Effects

	Coefficient	*t-value*	*S.E.*	*p-value*
Memory Success at Day 2				
Learning Condition	0.0001	1.0232	0.0001	0.3062
Relatedness	0.0002*	2.1957	0.0001	0.0281
Lure Strength - Weak Lures	0.00004	0.6908	0.00005	0.4897
Lure Strength - Moderate Lures	0.0002*	2.3437	0.00009	0.0191
Lure Strength - Strong Lures	0.0010***	6.1001	0.0002	<0.0001
Relatedness x Lure Strength - Weak Lures	-0.00009	-1.2492	0.00007	0.2116
Relatedness x Lure Strength - Moderate Lures	-0.0001	-0.8615	0.0001	0.389
Relatedness x Lure Strength - Strong Lures	0.00009	0.4255	0.0002	0.6705
Learning Condition x Lure Strength - Weak Lures	-0.0002**	-2.8405	0.00007	0.0045
Learning Condition x Lure Strength - Moderate Lures	0.000006	0.0485	0.0001	0.9613
Learning Condition x Lure Strength - Strong Lures	-0.0002	-1.0528	0.0002	0.2924
Learning Condition x Relatedness	-0.0002*	-2.3051	0.00007	0.0212
Num.Obs.	248508			
AIC	-1747564.2			
BIC	-1747355.7			
ICC	0.01			
RMSE	0.007			

Random Effects

	Variable 1	Variable 2	Variance	Std. Dev
PTID	(Intercept)		4.78E-07	0.00069123
	Relatedness		7.26E-07	0.00085178
	Learning Condition		6.17E-07	0.00078558
	(Intercept)	Relatedness	-2.86E-07	-0.4856958
	(Intercept)	Learning Condition	-3.58E-07	-0.6593156
	Relatedness	Learning Condition	2.16E-07	0.32304773
Residual			5.15E-05	0.00717846

Supplementary Table 3: Fixed and random effects for LMMs investigating pairwise change of to-be-learned pairs and semantic lures. All t tests are two-tailed and no corrections are made for multiple comparisons.

Pair Type

Fixed Effects

	Coefficient	*t-value*	*S.E.*	*p-value*
Pair Type - Learned, Correct at Day 2	0.0004	3.1121	0.0001	0.0021
Pair Type - Learned, Incorrect at Day 2	0.00007	0.5465	0.0001	0.5852
Num.Obs.	240			
AIC	-2637.4			
BIC	-2620			
ICC	0.2			
RMSE	0.0007			

Random Effects

	Variable	*Variance*	*Std. Dev*
PTID	(Intercept)	1.78E-07	0.00042243
Residual		6.37E-07	0.00079788

Supplementary Table 4: Fixed and random effects for LMMs investigating pairwise change depending on whether pair was recalled correctly at Day 2 and whether it was designated as a to-be-learned pair vs semantic lure
All t tests are two-tailed and no corrections are made for multiple comparisons.

	**Pairwise Change in Similarity of To-Be-Learned Pairs** *Fixed Effects*				**Pairwise Change in Similarity of Semantic Lures** *Fixed Effects*			
	Coefficient	*t-value*	*S.E.*	*p-value*	*Coefficient*	*t-value*	*S.E.*	*p-value*
Memory Success at Day 2	0.0003*	1.965	0.0002	0.0499				
Learning Condition	0.00006	0.3933	0.0002	0.6942	0.0001	1.0232	0.0001	0.3062
Relatedness	-0.0002	-1.3161	0.0002	0.1887	0.0002*	2.1957	0.0001	0.0281
Lure Strength - Weak Lures					0.00004	0.6908	0.00005	0.4897
Lure Strength - Moderate Lures					0.0002*	2.3437	0.00009	0.0191
Lure Strength - Strong Lures					0.0010***	6.1001	0.0002	<0.0001
Relatedness x Lure Strength - Weak Lures					-0.00009	-1.2492	0.00007	0.2116
Relatedness x Lure Strength - Moderate Lures					-0.0001	-0.8615	0.0001	0.389
Relatedness x Lure Strength - Strong Lures					0.00009	0.4255	0.0002	0.6705
Learning Condition x Lure Strength - Weak Lures					-0.0002**	-2.8405	0.00007	0.0045
Learning Condition x Lure Strength - Moderate Lures					0.000006	0.0485	0.0001	0.9613
Learning Condition x Lure Strength - Strong Lures					-0.0002	-1.0528	0.0002	0.2924
Learning Condition x Relatedness					-0.0002*	-2.3051	0.00007	0.0212
Num.Obs.	605				248508			
AIC	-5685.6				-1747564.2			
BIC	-5659.2				-1747355.7			
ICC	0.1				0.01			
RMSE	0.002				0.007			

Random Effects (To-Be-Learned Pairs)

	Variable	Variance	Std. Dev
PTID	(Intercept)	4.89E-07	0.00069945
Residual		3.96E-06	0.00198879

Random Effects (Semantic Lures)

	Variable 1	Variable 2	Variance	Std. Dev
PTID	(Intercept)		4.78E-07	0.00069123
	Relatedness		7.26E-07	0.00085178
	Learning Condition		6.17E-07	0.00078558
	(Intercept)	Relatedness	-2.86E-07	-0.4856958
	(Intercept)	Learning Condition	-3.58E-07	-0.6593156
	Relatedness	Learning Condition	2.16E-07	0.32304773
Residual			5.15E-05	0.00717846

Supplementary Table 5: Fixed and random effects for LMMs investigating pairwise change of to-be-learned pairs and semantic lures.
All t tests are two-tailed and no corrections are made for multiple comparisons.

Asymmetry

Fixed Effects

	Coefficient	*t-value*	*S.E.*	*p-value*
Memory Success at Day 2	-0.0059	-0.3988	0.0148	0.6902
Learning Condition	0.0002	0.0118	0.0177	0.9906
Relatedness	0.0356	2.4146	0.0147	0.0161
Num.Obs.	605			
AIC	-257.7			
BIC	-222.5			
ICC	0.1			
RMSE	0.17			

Random Effects

	Variable 1	Variable 2	Variance	Std. Dev.
PTID	(Intercept)		0.00021896	0.01479713
	Learning Condition		0.00754632	0.08686956
	(Intercept)	Learning Condition	0.00070642	0.54956229
Residual			0.03256754	0.18046478

Supplementary Table 6: Fixed and random effects for LMM investigating asymmetry
All t tests are two-tailed and no corrections are made for multiple comparisons.

Change Across Learning

Fixed Effects

	Coefficient	*t-value*	*S.E.*	*p-value*
Memory Success at Day 2	-0.078	-2.6604	0.0293	0.0079
Learning Condition	-0.0234	-0.7893	0.0296	0.4301
Word Position	-0.0071	-0.2384	0.0299	0.8116
Relatedness	-0.0309	-1.1301	0.0274	0.2586
Memory Success at Day 2 x Learning Condition	0.0494	1.258	0.0393	0.2086
Memory Success at Day 2 x Position	0.0995	2.5814	0.0385	0.01
Memory Success at Day 2 x Relatedness	0.077	1.9863	0.0387	0.0472
Learning Condition x Word Position	0.0249	0.7673	0.0324	0.4431
Learning Condition x Relatedness	0.0296	0.9085	0.0325	0.3638
Word Position x Relatedness	0.0438	1.3558	0.0323	0.1754
Memory Success at Day 2 x Learning Condition x Word Position	-0.0329	-0.7266	0.0452	0.4676
Memory Success at Day 2 x Learning Condition x Relatedness	-0.0583	-1.2841	0.0454	0.1994
Memory Success at Day 2 x Word Position x Relatedness	-0.1179	-2.6071	0.0452	0.0092
Num.Obs.	1210			
AIC	-164			
BIC	-57			
ICC	0.3			
RMSE	0.18			

Random Effects

	Variable 1	Variable 2	Variance	Std. Dev
PTID	(Intercept)		0.01523587	0.12343366
	Word Position		0.01122176	0.10593279
	Learning Condition		0.00499679	0.07068795
	(Intercept)	Word Position	-0.005304	-0.4056352
	(Intercept)	Learning Condition	-0.0013259	-0.1519599
	Word Position	Learning Condition	0.00370919	0.49534014
Residual			0.03841899	0.19600763

Difference in Correlation to Word2Vec (Final - Init)

Fixed Effects

	Coefficient	*t-value*	*S.E.*	*p-value*
Memory Success at Day 2	-0.0018	-0.1975	0.0093	0.8434
Learning Condition	0.0207	2.2159	0.0093	0.0269
Word Position	0.0009	0.1002	0.0093	0.9202
Relatedness	-0.0058	-0.6275	0.0093	0.5304
Num.Obs.	1210			
AIC	-881.5			
BIC	-845.8			
ICC	0.05			
RMSE	0.16			

Random Effects

	Variable	Variance	Std. Dev
PTID	(Intercept)	0.00134274	0.03664345
Residual		0.02616052	0.16174213

Supplementary Table 7: Fixed and random effects for LMMs investigating overall change in semantic space across learning and difference in change relative to normative semantic space
All t tests are two-tailed and no corrections are made for multiple comparisons.

Impact on Subsequent Behavior

Fixed Effects

	Coefficient	*t-value*	*S.E.*	*p-value*
Learning Condition	1.1809	6.3238	0.1867	<0.0001
Relatedness	-1.7256	-10.5724	0.1632	<0.0001
Change of Cue	-0.4286	-2.4531	0.1747	0.0142
Change of Target	0.1215	0.7137	0.1702	0.4754
Change in Cue Correlation to Word2vec	-0.1566	-0.7601	0.206	0.4472
Change in Target Correlation to Word2vec	-0.1818	-0.852	0.2134	0.3942
Asymmetry Value	-0.0111	-0.0588	0.1884	0.9532
Relatedness x Learning Condition	0.6167	2.4537	0.2513	0.0141
Relatedness x Change of Cue	0.1676	0.6562	0.2555	0.5117
Relatedness x Change of Target	-0.1509	-0.609	0.2478	0.5426
Relatedness x Change in Cue Correlation to Word2vec	0.4085	1.3245	0.3084	0.1853
Relatedness x Change in Target Correlation to Word2vec	0.155	0.5005	0.3097	0.6167
Relatedness x Asymmetry Value	0.027	0.0946	0.2852	0.9246
Learning Condition x Change of Cue	0.0391	0.132	0.2964	0.895
Learning Condition x Change of Target	0.2558	0.8572	0.2984	0.3913
Learning Condition x Change in Cue Correlation to Word2vec	-0.2701	-0.7493	0.3604	0.4537
Learning Condition x Change in Target Correlation to Word2vec	0.4831	1.2923	0.3738	0.1962
Learning Condition x Asymmetry Value	-0.2636	-0.7977	0.3304	0.425
Relatedness x Learning Condition x Change of Cue	0.3182	0.7712	0.4127	0.4406
Relatedness x Learning Condition x Change of Target	-0.8631	-2.0745	0.416	0.038
Relatedness x Learning Condition x Change in Cue Correlation to Word2vec	0.4297	0.823	0.5222	0.4105
Relatedness x Learning Condition x Change in Target Correlation to Word2vec	-0.1218	-0.2325	0.5238	0.8162
Relatedness x Learning Condition x Asymmetry Value	0.2818	0.5972	0.4718	0.5504
Num.Obs.	3902			
AIC	4430.3			
BIC	4618.4			
ICC	0.1			
RMSE	0.42			

Random Effects

	Variable 1	Variable 2	Variance	Std. Dev
PTID	(Intercept)		3.90E-07	0.00062453
	Learning Condition		4.11E-07	0.00064075
	Relatedness		2.94E-07	0.00054253
	(Intercept)	Learning Condition	-1.70E-07	-0.4237948
	(Intercept)	Relatedness	-9.88E-08	-0.2915987
	Learning Condition	Relatedness	-3.26E-08	-0.0938087
Residual			3.66E-05	0.00604818

Supplementary Table 8: Fixed and random effects for LMMs investigating behavioral relevance of representational change
All t tests are two-tailed and no corrections are made for multiple comparisons.

	Intercept	Pair Type - Learned, Correct at Day 2	Pair Type - Learned, Incorrect at Day 2
Intercept	1.02E-08	-7.96E-09	-7.96E-09
Pair Type - Learned, Correct at Day 2	-7.96E-09	1.59E-08	7.96E-09
Pair Type - Learned, Incorrect at Day 2	-7.96E-09	7.96E-09	1.59E-08

Supplementary Table 9: Variance/Covariance matrix for LMM investigating pairwise change depending on whether pair was correctly recalled at final test and whether it was a semantic lure

	(Intercept)	Learning Condition	Relatedness	Memory Success at Day 2
(Intercept)	3.21E-08	-1.20E-08	-1.35E-08	-1.35E-08
Learning Condition	-1.20E-08	2.63E-08	-3.63E-10	-1.02E-09
Relatedness	-1.35E-08	-3.63E-10	2.63E-08	9.99E-10
Memory Success at Day 2	-1.35E-08	-1.02E-09	9.99E-10	2.64E-08

Supplementary Table 10: Variance/Covariance matrix for LMM investigating pairwise change

	(Intercept)	Lure Strength - Weak Lure	Lure Strength - Moderate Lure	Lure Strength - Strong Lure	Relatedness	Learning Condition	Lure Strength - Weak Lure x Relatedness	Lure Strength - Moderate Lure x Relatedness	Lure Strength - Strong Lure x Relatedness	Lure Strength - Weak Lure x Learning Condition	Lure Strength - Moderate Lure x Learning Condition	Lure Strength - Strong Lure x Learning Condition	Relatedness x Learning Condition
(Intercept)	6.99E-09	-8.57E-10	-8.88E-10	-8.49E-10	-4.41E-09	-5.43E-09	4.56E-10	4.47E-10	4.27E-10	7.09E-10	7.58E-10	6.98E-10	6.51E-10
Lure Strength - Weak Lure	-8.57E-10	2.64E-09	8.66E-10	8.53E-10	4.87E-10	7.03E-10	-1.40E-09	-4.69E-10	-4.60E-10	-2.18E-09	-7.07E-10	-6.90E-10	-3.87E-11
Lure Strength - Moderate Lure	-8.88E-10	8.66E-10	8.31E-09	8.81E-10	5.35E-10	7.43E-10	-4.72E-10	-4.13E-09	-4.57E-10	-7.07E-10	-7.19E-09	-7.20E-10	-1.07E-10
Lure Strength - Strong Lure	-8.49E-10	8.53E-10	8.81E-10	2.82E-08	4.47E-10	6.79E-10	-4.71E-10	-4.70E-10	-1.45E-08	-6.88E-10	-7.17E-10	-2.32E-08	5.12E-11
Relatedness	-4.41E-09	4.87E-10	5.35E-10	4.47E-10	1.24E-08	3.32E-09	-1.52E-09	-1.49E-09	-1.43E-09	5.23E-12	-1.00E-10	5.85E-11	-2.44E-09
Learning Condition	-5.43E-09	7.03E-10	7.43E-10	6.79E-10	3.32E-09	9.58E-09	9.55E-11	1.22E-10	1.42E-10	-1.40E-09	-1.50E-09	-1.39E-09	-1.29E-09
Lure Strength - Weak Lure x Relatedness	4.56E-10	-1.40E-09	-4.72E-10	-4.71E-10	-1.52E-09	9.55E-11	5.00E-09	1.60E-09	1.54E-09	-2.24E-10	-8.12E-11	-7.79E-11	-8.93E-11
Lure Strength - Moderate Lure x Relatedness	4.47E-10	-4.69E-10	-4.13E-09	-4.70E-10	-1.49E-09	1.22E-10	1.60E-09	1.72E-08	1.58E-09	-7.53E-11	-5.24E-10	-8.98E-11	-1.56E-10
Lure Strength - Strong Lure x Relatedness	4.27E-10	-4.60E-10	-4.57E-10	-1.45E-08	-1.43E-09	1.42E-10	1.54E-09	1.58E-09	4.79E-08	-7.97E-11	-8.85E-11	-1.77E-09	-1.55E-10
Lure Strength - Weak Lure x Learning Condition	7.09E-10	-2.18E-09	-7.07E-10	-6.88E-10	5.23E-12	-1.40E-09	-2.24E-10	-7.53E-11	-7.97E-11	4.25E-09	1.41E-09	1.37E-09	1.20E-10
Lure Strength - Moderate Lure x Learning Condition	7.58E-10	-7.07E-10	-7.19E-09	-7.17E-10	-1.00E-10	-1.50E-09	-8.12E-11	-5.24E-10	-8.85E-11	1.41E-09	1.33E-08	1.43E-09	3.15E-10
Lure Strength - Strong Lure x Learning Condition	6.98E-10	-6.90E-10	-7.20E-10	-2.32E-08	5.85E-11	-1.39E-09	-7.79E-11	-8.98E-11	-1.77E-09	1.37E-09	1.43E-09	4.26E-08	2.59E-11
Relatedness x Learning Condition	6.51E-10	-3.87E-11	-1.07E-10	5.12E-11	-2.44E-09	-1.29E-09	-8.93E-11	-1.56E-10	-1.55E-10	1.20E-10	3.15E-10	2.59E-11	4.50E-09

Supplementary Table 11: Variance/Covariance matrix for LMM investigating pairwise change of semantic lures

	(Intercept)	Memory Success at Day 2	Learning Condition	Word Position	Relatedness	Memory Success at Day 2 x Learning Condition	Memory Success at Day 2 x Word Position	Learning Condition x Word Position	Memory Success at Day 2 x Relatedness	Learning Condition x Relatedness	Word Position x Relatedness	Memory Success at Day 2 x Learning Condition x Word Position	Memory Success at Day 2 x Learning Condition x Relatedness	Memory Success at Day 2 x Word Position x Relatedness
(Intercept)	0.0006267	-0.000436243	-0.000382471	-0.000444172	-0.0003799	0.000365938	0.000377913	0.000237042	0.000379709	0.00024743	0.00026513	-0.000237124	-0.00024738	-0.000265049
Memory Success at Day 2	-0.000436	0.000858736	0.000365882	0.000377912	0.00037989	-0.00073063	-0.000742661	-0.000236956	-0.000739423	-0.0002475	-0.0002652	0.000486216	0.000487544	0.000504358
Learning Condition	-0.000382	0.000365882	0.000876769	0.000284132	0.00024141	-0.000814278	-0.000237737	-0.000523614	-0.000241884	-0.0005521	1.12E-05	0.000523553	0.000552623	-1.11E-05
Word Position	-0.000444	0.000377912	0.000284132	0.000895713	0.00026515	-0.000237847	-0.00075552	-0.000473965	-0.000265054	-1.02E-06	-0.00053	0.000474124	1.00E-06	0.000529865
Relatedness	-0.00038	0.000379891	0.000241408	0.000265153	0.00074882	-0.000241485	-0.00026523	1.24E-05	-0.000748668	-0.0004879	-0.0005228	-1.23E-05	0.000487856	0.000522764
Memory Success at Day 2 x Learning Condition	0.0003659	-0.00073063	-0.000814278	-0.000237847	-0.0002415	0.001543663	0.000486994	0.000523442	0.000481074	0.00055216	-1.11E-05	-0.001021735	-0.001032952	1.27E-05
Memory Success at Day 2 x Word Position	0.0003779	-0.000742661	-0.000237737	-0.00075552	-0.0002652	0.000486994	0.001485015	0.000473793	0.000504245	1.08E-06	0.00053015	-0.000972306	-1.09E-06	-0.001008482
Learning Condition x Word Position	0.000237	-0.000236956	-0.000523614	-0.000473965	1.24E-05	0.000523442	0.000473793	0.001049614	-1.21E-05	-5.38E-07	-2.50E-05	-0.001049269	4.32E-07	2.45E-05
Memory Success at Day 2 x Relatedness	0.0003797	-0.000739423	-0.000241884	-0.000265054	-0.0007487	0.000481074	0.000504245	-1.21E-05	0.001501132	0.00048807	0.00052274	1.40E-05	-0.00099079	-0.001022343
Learning Condition x Relatedness	0.0002474	-0.000247462	-0.000552103	-1.02E-06	-0.0004879	0.000552159	1.08E-06	-5.38E-07	0.000488069	0.00105948	1.72E-06	4.24E-07	-0.001060132	-1.70E-06
Word Position x Relatedness	0.0002651	-0.000265207	1.12E-05	-0.000530002	-0.0005228	-1.11E-05	0.000530153	-2.50E-05	0.000522736	1.72E-06	0.00104543	2.47E-05	-1.72E-06	-0.001045295
Memory Success at Day 2 x Learning Condition x Word Position	-0.000237	0.000486216	0.000523553	0.000474124	-1.23E-05	-0.001021735	-0.000972306	-0.001049269	1.40E-05	4.24E-07	2.47E-05	0.002045633	-2.50E-07	-2.77E-05
Memory Success at Day 2 x Learning Condition x Relatedness	-0.000247	0.000487544	0.000552623	1.00E-06	0.00048786	-0.001032952	-1.09E-06	4.32E-07	-0.00099079	-0.0010601	-1.72E-06	-2.50E-07	0.002062374	2.42E-06
Memory Success at Day 2 x Word Position x Relatedness	-0.000265	0.000504358	-1.11E-05	0.000529865	0.00052276	1.27E-05	-0.001008482	2.45E-05	-0.001022343	-1.70E-06	-0.0010453	-2.77E-05	2.42E-06	0.002043924

Supplementary Table 12: Variance/Covariance matrix for LMM investigating change in overall semantic space across learning

	(Intercept)	Learning Condition	Word Position	Relatedness	Memory Success at Day 2
(Intercept)	0.0001243	-3.98E-05	-4.32E-05	-4.45E-05	-4.46E-05
Learning Condition	-3.98E-05	8.70E-05	1.12E-22	-1.19E-06	-3.33E-06
Word Position	-4.32E-05	1.12E-22	8.65E-05	-8.17E-21	-3.08E-22
Relatedness	-4.45E-05	-1.19E-06	-8.17E-21	8.69E-05	3.28E-06
Memory Success at Day 2	-4.46E-05	-3.33E-06	-3.08E-22	3.28E-06	8.71E-05

Supplementary Table 13: Variance/Covariance matrix for LMM investigating difference in change to normative semantic space across learning

	Intercept	Learning Condition	Relatedness	Memory Success at Day 2
Intercept	0.000217	-8.98E-05	-0.00011112	-0.000111484
Learning Condition	-8.98E-05	0.000311695	-2.97E-06	-8.81E-06
Relatedness	-0.0001111	-2.97E-06	0.000216843	8.30E-06
Memory Success at Day 2	-0.0001115	-8.81E-06	8.30E-06	0.000217597

Supplementary Table 14: Variance/Covariance matrix for LMM investigating asymmetrical change

	(Intercept)	Change in Cue Representation	Relatedness	Learning Condition	Change in Target Representation	Difference in Cue Correlation to Word2Vec	Difference in Target Correlation to Word2Vec	Representational Asymmetry	Change in Cue Representation x Relatedness	Change in Cue Representation x Learning Condition	Relatedness x Learning Condition	Relatedness x Change in Target Representation	Learning Condition x Change in Target Representation	Learning Condition x Difference in Cue Correlation to Word2Vec	Relatedness x Difference in Cue Correlation to Word2Vec	Learning Condition x Difference in Target Correlation to Word2Vec
(Intercept)	0.01518845	-0.007723937	-0.009779424	-0.011799234	-0.008785857	-0.000650711	-0.00066305	0.000316012	0.007366358	0.007516895	0.009186777	0.008436408	0.008491874	0.000605942	0.000557493	0.000702616
Change in Cue Representation	-0.0077239	0.030523804	0.007430425	0.007559522	-0.005609589	-0.000591349	0.000344237	-0.00241966	-0.029821712	-0.030070326	-0.007300384	0.005985763	0.005811064	0.000732208	0.000694006	-0.000490636
Relatedness	-0.0097794	0.007430425	0.026640988	0.008620568	0.008488977	0.00059349	0.000784678	-0.000270089	-0.01858105	-0.007200741	-0.022416194	-0.019774127	-0.008390938	-0.000489842	-0.000180018	-0.000800031
Learning Condition	-0.0117992	0.007559522	0.008620568	0.034869045	0.008488292	0.000590583	0.000514069	-0.000246237	-0.00704658	-0.023319875	-0.029761238	-0.008060896	-0.02590338	-0.004208269	-0.000413467	-0.003926201
Change in Target Representation	-0.0087859	-0.005609589	0.008488977	0.008488292	0.028966518	0.002193885	0.000571517	0.002122998	0.006053586	0.005769183	-0.008184738	-0.028383265	-0.028437818	-0.00224762	-0.0021568	-0.000606589
Difference in Cue Correlation to Word2Vec	-0.0006507	-0.000591349	0.00059349	0.000590583	0.002193885	0.042452509	0.002105726	0.001286379	0.000720689	0.000518112	-0.000497024	-0.002178681	-0.002255235	-0.04233158	-0.042181838	-0.001882816
Difference in Target Correlation to Word2Vec	-0.0006631	0.000344237	0.000784678	0.000514069	0.000571517	0.002105726	0.045545429	-0.002195511	-0.000480145	-0.000490703	-0.000584664	-0.000734102	-0.0004466	-0.001957084	-0.002036576	-0.045248961
Representational Asymmetry	0.00031601	-0.00241966	-0.000270089	-0.000246237	0.002122998	0.001286379	-0.002195511	0.03551196	0.002444785	0.002406631	0.000177627	-0.002221796	-0.002139532	-0.00135874	-0.001170406	0.002229224
Change in Cue Representation x Relatedness	0.00736636	-0.029821712	-0.01858105	-0.00704658	0.006053586	0.000720689	-0.000480145	0.002444785	0.06526469	0.029271028	0.017886134	-0.006819009	-0.006099814	-0.000944551	-0.000699499	0.000569052
Change in Cue Representation x Learning Condition	0.0075169	-0.030070326	-0.007200741	-0.023319875	0.005769183	0.000518112	-0.000490703	0.002406631	0.029271028	0.087871564	0.022539029	-0.006189912	-0.017844609	0.001156134	-0.000644134	-0.000383431
Relatedness x Learning Condition	0.00918678	-0.007300384	-0.022416194	-0.029761238	-0.008184738	-0.000497024	-0.000584664	0.000177627	0.017886134	0.022539029	0.063167163	0.018967478	0.025318734	0.003978289	-7.52E-05	0.004052072
Relatedness x Change in Target Representation	0.00843641	0.005985763	-0.019774127	-0.008060896	-0.028383265	-0.002178681	-0.000734102	-0.002221796	-0.006819009	-0.006189912	0.018967478	0.061418099	0.027782213	0.002245665	0.001182701	0.000792375
Learning Condition x Change in Target Representation	0.00849187	0.005811064	-0.008390938	-0.02590338	-0.028437818	-0.002255235	-0.0004466	-0.002139532	-0.006099814	-0.017844609	0.025318734	0.027782213	0.089015474	0.005954925	0.00214308	0.006522489
Learning Condition x Difference in Cue Correlation to Word2Vec	0.00060594	0.000732208	-0.000489842	-0.004208269	-0.00224762	-0.04233158	-0.001957084	-0.00135874	-0.000944551	0.001156134	0.003978289	0.002245665	0.005954925	0.129888645	0.042071543	0.005346271
Relatedness x Difference in Cue Correlation to Word2Vec	0.00055749	0.000694006	-0.000180018	-0.000413467	-0.0021568	-0.042181838	-0.002036576	-0.001170406	-0.000699499	-0.000644134	-7.52E-05	0.001182701	0.00214308	0.042071543	0.095122117	0.001758959
Learning Condition x Difference in Target Correlation to Word2Vec	0.00070262	-0.000490636	-0.000800031	-0.003926201	-0.000606589	-0.001882816	-0.045248961	0.002229224	0.000569052	-0.000383431	0.004052072	0.000792375	0.006522489	0.005346271	0.001758959	0.139714596
Relatedness x Difference in Target Correlation to Word2Vec	0.00064018	-0.000482916	0.000186139	-0.000435035	-0.000478109	-0.002021341	-0.045127601	0.002316869	0.001184457	0.000662877	-0.000400739	-0.00111536	0.000370719	0.00176061	0.004153938	0.044740894
Relatedness x Representational Asymmetry	-0.0002932	0.002485483	0.000456214	0.000175872	-0.002213238	-0.001414743	0.002333175	-0.035404325	-0.002592631	-0.002454679	-0.000252076	0.000780618	0.002123887	0.001558519	0.001889195	-0.002334456
Learning Condition x Representational Asymmetry	-0.0003188	0.002311734	0.000156764	0.00032228	-0.002126638	-0.001097128	0.002498802	-0.035470124	-0.002281957	-0.005869572	2.28E-05	0.002238519	0.006138851	0.008670705	0.0009494	-0.005082047
Change in Cue Representation x Relatedness x Learning Condition	-0.0071657	0.029388229	0.018077023	0.02228391	-0.006155308	-0.000651232	0.000626479	-0.002448365	-0.064141122	-0.086505147	-0.047545979	0.00745659	0.018836055	-0.00077684	0.000931844	0.000454415
Relatedness x Learning Condition x Change in Target Representation	-0.0080855	-0.006163814	0.019249329	0.024188952	0.027852896	0.00214096	0.000506254	0.002257748	0.007637578	0.019046147	-0.05392883	-0.059848072	-0.0872513	-0.005969964	-0.001234421	-0.006834098
Relatedness x Learning Condition x Difference in Cue Correlation to Word2Vec	-0.0004881	-0.000829688	0.000142616	0.003760045	0.002242201	0.042027997	0.0018414	0.001257506	0.000918656	-0.000631705	-0.006598327	-0.001403375	-0.005961061	-0.129265348	-0.094668833	-0.004823411
Relatedness x Learning Condition x Difference in Target Correlation to Word2Vec	-0.000688	0.000632057	-0.000127064	0.003999122	0.000591326	0.001816928	0.044852825	-0.002376653	-0.001222105	0.000102098	-0.003652059	0.000974026	-0.006623178	-0.004742923	-0.00353158	-0.138993505
Relatedness x Learning Condition x Representational Asymmetry	0.00028579	-0.002359023	-0.000293195	-0.000157047	0.002168758	0.001263077	-0.002595479	0.03535982	0.002447443	0.006016905	-0.001663838	-0.000735857	-0.006024175	-0.008651687	-0.001840383	0.005136568

Supplementary Table 15: Variance/Covariance matrix for LMM investigating relevance of representational change to memory at Day 2

Relatedness x Difference in Target Correlation to Word2Vec	Relatedness x Representational Asymmetry	Learning Condition x Representational Asymmetry	Change in Cue Representation x Relatedness x Learning Condition	Relatedness x Learning Condition x Change in Target Representation	Relatedness x Learning Condition x Difference in Cue Correlation to Word2Vec	Relatedness x Learning Condition x Difference in Target Correlation to Word2Vec	Relatedness x Learning Condition x Representational Asymmetry
0.000640182	-0.000293236	-0.000318803	-0.007165736	-0.008085511	-0.000488125	-0.000687988	0.000285787
-0.000482916	0.002485483	0.002311734	0.029388229	-0.006163814	-0.000829688	0.000632057	-0.002359023
0.000186139	0.000456214	0.000156764	0.018077023	0.019249329	0.000142616	-0.000127064	-0.000293195
-0.000435035	0.000175872	0.00032228	0.02228391	0.024188952	0.003760045	0.003999122	-0.000157047
-0.000478109	-0.002213238	-0.002126638	-0.006155308	0.027852896	0.002242201	0.000591326	0.002168758
-0.002021341	-0.001414743	-0.001097128	-0.000651232	0.00214096	0.042027997	0.001816928	0.001263077
-0.045127601	0.002333175	0.002498802	0.000626479	0.000506254	0.0018414	0.044852825	-0.002595479
0.002316869	-0.035404325	-0.035470124	-0.002448365	0.002257748	0.001257506	-0.002376653	0.03535982
0.001184457	-0.002592631	-0.002281957	-0.064141122	0.007637578	0.000918656	-0.001222105	0.002447443
0.000662877	-0.002454679	-0.005869572	-0.086505147	0.019046147	-0.000631705	0.000102098	0.006016905
-0.000400739	-0.000252076	2.28E-05	-0.047545979	-0.05392883	-0.006598327	-0.003652059	-0.001663838
-0.00111536	0.000780618	0.002238519	0.00745659	-0.059848072	-0.001403375	0.000974026	-0.000735857
0.000370719	0.002123887	0.006138851	0.018836055	-0.0872513	-0.005961061	-0.006623178	-0.006024175
0.00176061	0.001558519	0.008670705	-0.00077684	-0.005969964	-0.129265348	-0.004742923	-0.008651687
0.004153938	0.001889195	0.0009494	0.000931844	-0.001234421	-0.094668833	-0.00353158	-0.001840383
0.044740894	-0.002334456	-0.005082047	0.000454415	-0.006834098	-0.004823411	-0.138993505	0.005136568
0.095901503	-0.001262825	-0.002641187	-0.001450542	0.001056773	-0.0037508	-0.095213788	0.001305006
-0.001262825	0.081356707	0.035326317	0.002571314	-0.0008253	-0.002016925	0.001470084	-0.081225436
-0.002641187	0.035326317	0.109155852	0.005738083	-0.006221695	-0.008754021	0.005580281	-0.108875043
-0.001450542	0.002571314	0.005738083	0.170303314	-0.026832052	0.007367411	0.002868466	-0.00923382
0.001056773	-0.0008253	-0.006221695	-0.026832052	0.173076925	0.004502992	0.003368141	0.008629733
-0.0037508	-0.002016925	-0.008754021	0.007367411	0.004502992	0.272668046	0.006963147	0.006044928
-0.095213788	0.001470084	0.005580281	0.002868466	0.003368141	0.006963147	0.274356241	0.00130354
0.001305006	-0.081225436	-0.108875043	-0.00923382	0.008629733	0.006044928	0.00130354	0.222640825
