# Supplementary material for: Behavioral representational similarity analysis reveals how episodic learning is influenced by and reshapes semantic memory
Source: Nat Commun. 2023 Nov 20;14:7548. doi: 10.1038/s41467-023-42770-w (PMC10662157; doi:10.1038/s41467-023-42770-w)
Supplement: Supplementary file 3 — Reporting Summary [file 41467_2023_42770_MOESM3_ESM.pdf]

nature portfolio

Corresponding author(s): Catherine Walsh

Last updated by author(s): Oct 11, 2023

Reporting Summary

Nature Portfolio wishes to improve the reproducibility of the work that we publish. This form provides structure for consistency and transparency in reporting. For further information on Nature Portfolio policies, see our Editorial Policies and the Editorial Policy Checklist.

Statistics

For all statistical analyses, confirm that the following items are present in the figure legend, table legend, main text, or Methods section.

n/a	Confirmed	
☐	☒	The exact sample size (n) for each experimental group/condition, given as a discrete number and unit of measurement
☐	☒	A statement on whether measurements were taken from distinct samples or whether the same sample was measured repeatedly
☐	☒	The statistical test(s) used AND whether they are one- or two-sided<br>*Only common tests should be described solely by name; describe more complex techniques in the Methods section.*
☐	☒	A description of all covariates tested
☐	☒	A description of any assumptions or corrections, such as tests of normality and adjustment for multiple comparisons
☐	☒	A full description of the statistical parameters including central tendency (e.g. means) or other basic estimates (e.g. regression coefficient) AND variation (e.g. standard deviation) or associated estimates of uncertainty (e.g. confidence intervals)
☐	☒	For null hypothesis testing, the test statistic (e.g. F, t, r) with confidence intervals, effect sizes, degrees of freedom and P value noted<br>*Give P values as exact values whenever suitable.*
☒	☐	For Bayesian analysis, information on the choice of priors and Markov chain Monte Carlo settings
☐	☒	For hierarchical and complex designs, identification of the appropriate level for tests and full reporting of outcomes
☐	☒	Estimates of effect sizes (e.g. Cohen's d, Pearson's r), indicating how they were calculated

Our web collection on statistics for biologists contains articles on many of the points above.

Software and code

Policy information about availability of computer code

Data collection	Data were collected using custom experimental code created using lab.js (https://labjs.felixhenninger.com/). The experiment was hosted on a local server and subjects were recruited using Prolific (https://www.prolific.co/) and through the UCLA SONA Undergraduate Participant Pool.
Data analysis	Data were analyzed using custom code written in R version 4.2.2 running under macOs Monterey 12.4 (aarch65-apple-darwin20 - 64 bit)with packages: effectsize (0.8.3), emmeans (1.8.3), varTestnlme (1.3.1), r2mlm (0.3.2), nlme (3.1-160), forcats (0.5.2), stringr (1.5.0), dplyr (1.1.0), purrr (1.0.1), readr (2.1.4), tidyr (1.3.0), tibble (3.2.1), ggplot2 (3.4.2), tidyverse (1.3.2), rstatix (0.7.1), reshape2 (1.4.4), lmerTest (3.1-3), lme4 (1.1-31), Matrix (1.5-0), Corrplot (0.92), ggpattern (1.0.1), easystats (0.6.0). All code necessary to reproduce analyses is fully available at https://osf.io/5q6th/ (DOI: (https://doi.org/10.17605/OSF.IO/5Q6TH).

For manuscripts utilizing custom algorithms or software that are central to the research but not yet described in published literature, software must be made available to editors and reviewers. We strongly encourage code deposition in a community repository (e.g. GitHub). See the Nature Portfolio guidelines for submitting code & software for further information.

Data

Policy information about availability of data

All manuscripts must include a data availability statement. This statement should provide the following information, where applicable:

- Accession codes, unique identifiers, or web links for publicly available datasets
- A description of any restrictions on data availability
- For clinical datasets or third party data, please ensure that the statement adheres to our policy

The raw behavioral data generated in this study have been deposited in the Open Science Framework at https://osf.io/5q6th/ (https://doi.org/10.17605/OSF.IO/5Q6TH). LSA cosine similarity data are available at http://www.lingexp.uni-tuebingen.de/z2/LSAspaces/. Pre-trained word2vec model is available at https://code.google.com/archive/p/word2vec/.

Research involving human participants, their data, or biological material

Policy information about studies with human participants or human data. See also policy information about sex, gender (identity/presentation), and sexual orientation and race, ethnicity and racism.

Reporting on sex and gender	Sex/gender were not considered in our study design; our results should be applicable regardless of sex/gender. Sex/gender were self-reported. We did not conduct any analyses on sex/gender as we opted for a within-subjects design.
Reporting on race, ethnicity, or other socially relevant groupings	We did not include analyses on race, ethnicity or other socially relevant groupings as we opted for a within-subjects design.
Population characteristics	See responses to Behavioural and social sciences study design section.
Recruitment	We collected recruited N = 80 subjects (29 male; mean age = 24.33, SD = 5.49) from Prolific (https://www.prolific.co/) and through the UCLA SONA Undergraduate Participant Pool. Participants from SONA were compensated with course credit, while participants from Prolific received monetary compensation. The two samples were not different on any key outcome measures, so we combined the samples for a final N = 80 dataset. We do not believe there are any selection biases in our sample.
Ethics oversight	This research was approved by the IRB of the University of California, Los Angeles.

Note that full information on the approval of the study protocol must also be provided in the manuscript.

Field-specific reporting

Please select the one below that is the best fit for your research. If you are not sure, read the appropriate sections before making your selection.

☐ Life sciences ☒ Behavioural & social sciences ☐ Ecological, evolutionary & environmental sciences

For a reference copy of the document with all sections, see nature.com/documents/nr-reporting-summary-flat.pdf

Behavioural & social sciences study design

All studies must disclose on these points even when the disclosure is negative.

Study description	This study was a quantitative within subject experiment. We investigated the effect of pre-existing semantic information and learning condition (i.e. testing vs restudying) on recall after an approximately 24 hour delay.
Research sample	We collected recruited N = 80 subjects (29 male; mean age = 24.33, SD = 5.49) from Prolific (https://www.prolific.co/) and through the UCLA SONA Undergraduate Participant Pool. We did not collect information about race and restricted our analyses to individuals with no current or ongoing mental health or neurological condition and whose first language was English. For the data collected from Prolific, we restricted our recruitment to adults (age 18-40) from the United States, Mexico and Canada. Data was not a representative sample. Data were collected online due to the COVID-19 pandemic. We included the aforementioned inclusion criteria to avoid any confounds of memory impairment due to age or psychiatric illness or insufficient understanding of semantic meaning of English words.
Sampling strategy	We used a random sampling approach. We conducted a power analysis prior to collecting data based on information from a previously published meta-analysis which suggested that our expected effects would be medium effect sizes. We anticipated that effects on representational similarity would be considerably smaller and there were no prior studies to anchor assumptions in, so we assumed a small effect size. With this in mind, we used the PANGEA (v0.2) app (https://jakewestfall.shinyapps.io/pangea) to determine that we would need 73 useable datasets to reach a power of 0.8 for our main interactions. We opted to aim for 80 datasets to ensure we were sufficiently powered to see this effect. Further information about our sampling strategy are available in our preregistration (https://osf.io/zrvxh) and in the Supplementary Methods of our manuscript.

Data collection	Data were collected online due to data collection occurring during the COVID-19 pandemic. The experimenter was not blind to experimental conditions or hypotheses but was not present during data collection. We instructed our participants be in a quiet room with no distractions and that they complete our study on a desktop computer. A survey was administered at the end of the experiment to confirm that there were no distractions present during the data collection.
Timing	Data collection began on 12/22/2020 and completed on 2/27/2021.
Data exclusions	We used relatively strict exclusion criteria given our data collection occurred online. These exclusion criteria were pre-registered (https://osf.io/zrvxh), are reported in our Supplementary Information and are listed below.<br><br>Fail to complete Day 2 within 28 hours of completing Day 1<br>• Fail to respond correctly to attention checks<br>• Have a median RT of less than 500 ms in the first learning session while making relatedness judgments<br>• Fail to show a difference between relatedness judgements on related and unrelated words<br>• Report that they believe their data should be excluded or report some sort of technical issue<br>• Report distraction of greater than 5 out of 7 on our distraction scale for either of the days, or report three or more distractions occurring on either day<br>• Do not perform a meaningful arrangement on one or more of the trials of the word arrangement task. Subjects who judged similarity based on lexical characteristics (i.e. the first letter of each word), or who randomly placed words on the page were excluded. Meaningfulness was judged by 3 independent raters and participants were excluded if 2 of 3 raters agreed that either pre or post learning arrangement was considered not meaningful.<br>• Do not have a sufficient performance on the memory tasks. For example, the participants will be excluded if any of the following are true:<br>o Those who fail to correctly retype three or more of the restudy trials on the initial learning day<br>o Those who fail to recall more than 25% of the tested associates during the initial learning session, or more than 25% across all pairs during the second session<br>• Explicitly report writing pairs down between sessions<br><br>We noted in our pre-registration that we would exclude subjects who reported rehearsing words between sessions, but we ultimately decided to include the 8 subjects who reported rehearsing, sessions, as we did not explicitly instruct participants not to rehearse and our survey question was not specific enough to determine the extent to which they rehearsed (i.e. it did not distinguish whether they spent hours rehearsing all word pairs, or just happened to spontaneously recall one or two of them).
Non-participation	We initially collected 262 participants (145 from SONA, 117 from Prolific). Of these, 183 (88 from SONA, 95 from Prolific) returned for the second session. We do not have any information why these subjects dropped out.
Randomization	Our design was within-subject, so subjects were not allocated to groups. Word pairs were randomly assigned to either the testing or restudying condition for each individual.

Reporting for specific materials, systems and methods

We require information from authors about some types of materials, experimental systems and methods used in many studies. Here, indicate whether each material, system or method listed is relevant to your study. If you are not sure if a list item applies to your research, read the appropriate section before selecting a response.

Materials & experimental systems

n/a	Involved in the study
☒	☐ Antibodies
☒	☐ Eukaryotic cell lines
☒	☐ Palaeontology and archaeology
☒	☐ Animals and other organisms
☒	☐ Clinical data
☒	☐ Dual use research of concern
☒	☐ Plants

Methods

n/a	Involved in the study
☒	☐ ChIP-seq
☒	☐ Flow cytometry
☒	☐ MRI-based neuroimaging

Plants

Seed stocks	*Report on the source of all seed stocks or other plant material used. If applicable, state the seed stock centre and catalogue number. If plant specimens were collected from the field, describe the collection location, date and sampling procedures.*
Novel plant genotypes	*Describe the methods by which all novel plant genotypes were produced. This includes those generated by transgenic approaches, gene editing, chemical/radiation-based mutagenesis and hybridization. For transgenic lines, describe the transformation method, the number of independent lines analyzed and the generation upon which experiments were performed. For gene-edited lines, describe the editor used, the endogenous sequence targeted for editing, the targeting guide RNA sequence (if applicable) and how the editor was applied.*
Authentication	*Describe any authentication procedures for each seed stock used or novel genotype generated. Describe any experiments used to assess the effect of a mutation and, where applicable, how potential secondary effects (e.g. second site T-DNA insertions, mosiacism, off-target gene editing) were examined.*
